# Number of Episodes Can Be Used as a Disease Activity Measure in Familial Mediterranean Fever

**DOI:** 10.3389/fped.2022.822473

**Published:** 2022-04-27

**Authors:** David Piskin, Zehra Serap Arici, Dilek Konukbay, Micol Romano, Balahan Makay, Nuray Ayaz, Yelda Bilginer, Roberta A. Berard, Hakan Poyrazoglu, Ozgur Kasapcopur, Ronald M. Laxer, Kathy Speechley, Erkan Demirkaya

**Affiliations:** ^1^Children’s Health Research Institute, Lawson Health Research Institute, London, ON, Canada; ^2^Department of Paediatrics and Epidemiology and Biostatistics, Schulich School of Medicine and Dentistry, University of Western Ontario, London, ON, Canada; ^3^Clinical Epidemiology, McMaster University, Hamilton, ON, Canada; ^4^Gulhane Faculty of Nursing, University of Health Sciences, Ankara, Turkey; ^5^Division of Paediatric Rheumatology, Department of Paediatrics, Schulich School of Medicine and Dentistry, University of Western Ontario, London, ON, Canada; ^6^Canadian Behcet’s and Autoinflammatory Diseases Center (CAN BE AID), University of Western Ontario, London, ON, Canada; ^7^Pediatric Rheumatology Unit, Dokuz Eylül University, ízmir, Turkey; ^8^Department of Pediatric Rheumatology, Istanbul University Medical School, Istanbul, Turkey; ^9^Pediatric Rheumatology Unit, Faculty of Medicine, Hacettepe University, Ankara, Turkey; ^10^Division of Rheumatology, Department of Pediatrics, Faculty of Medicine, Erciyes University, Kayseri, Turkey; ^11^Pediatric Rheumatology Unit, Cerrahpasa Medical Faculty, Istanbul University, Istanbul, Turkey; ^12^The Hospital for Sick Children, University of Toronto, Toronto, ON, Canada

**Keywords:** Familial Mediterranean fever, disease activity, quality of life, patient reported outcomes, convergent validity

## Abstract

**Objective:**

To evaluate the number of episodes in the past 12 months as an indicator of the overall disease activity status in Familial Mediterranean fever (FMF).

**Methods:**

In this cross-sectional study, patients were recruited from tertiary pediatric hospitals. Demographic data, main clinical symptoms of the episodes, treatment modalities, and genetic mutations were recorded. The patients were grouped as no episodes (Group 1), 1–4 episodes (Group 2), and more than 4 episodes (Group 3) according to the number of episodes in the past 12 months. The Pediatric Quality Life Inventory (PedsQL), the Children’s Depression Inventory (CDI), and the Wong-Baker FACES Pain Rating Scale (FACES) scores were compared between groups. Concurrent validity between the number of episodes and the patient-reported outcome measures (PROMs) was assessed using Spearman’s rank correlation coefficient (ρ).

**Results:**

A total of 239 patients were included. There were 74 patients (31%) in Group 1, 99 (41.4%) in Group 2, and 66 (27.6%) in Group 3. Groups were similar according to age, age at diagnosis, gender, consanguinity, family history, history of amyloidosis, clinical symptoms, and in terms of allele frequency (*p* > 0.05). According to PROMs completed by parents, moderate correlations were found between the number of episodes and the PedsQL score (ρ = −0.48; 95% *CI* = −0.58 to −0.35, *p* < 0.001) and between the number of episodes and the Wong-Baker FACES score (ρ = 0.47, 95% *CI* = 0.35–0.57, *p* < 0.001).

**Conclusion:**

The number of episodes was positively and moderately correlated with patient- and parent-reported outcomes in our cohort. The number of episodes in patients with FMF can be used as a single measure to assess disease activity.

## Introduction

Familial Mediterranean fever (FMF) (OMIM #249100) is the most common monogenic autoinflammatory disease (AID) characterized by recurring febrile episodes of 1–3 days accompanied by inflammation in the serous membranes causing peritonitis, pleuritis, or synovitis. Colchicine is the first-line treatment, and if a patient is resistant or a non-responder, biologics can be used to suppress episodes and systemic inflammation. The most devastating complication is amyloidosis in untreated and non-compliant patients with FMF. There are some phenotypic features associated with a severe disease course, such as the presence of one (or two) M694V mutations, ethnicity, and country of residence (1).

Monitoring disease activity in FMF is essential to measure the effectiveness of treatments, prevent complications, and quantify the effect of the disease on the overall health and quality of life (QoL). The importance of regular monitoring of disease activity was highlighted in the international recommendations for the management of FMF (2, 3). During the past decade, there were several attempts to develop instruments to measure disease severity, damage, and activity for FMF and other autoinflammatory diseases (AIDs) (4, 5). There are a few patient-reported outcome measures (PROMs) validated for use in FMF (6). The only available patient-reported tool to measure disease activity for FMF is the Autoinflammatory Disease Activity Index (AIDAI) (7), which was developed and validated by an international consortium (8), but its use in both research and clinical practice has been limited (9). It is challenging to complete a prospective diary for a long time, especially in adolescents with FMF. Except for the use reported in the publication describing its validation, there has been no uptake of the AIDAI as an outcome instrument related to the FMF.

We propose that the number of episodes could reasonably be used as an indicator of disease activity status in patients with FMF. We assessed the concurrent validity of the number of episodes as a feasible stand-alone measure of disease activity. To do so, we developed *a priori* predictions of the associations that the number of episodes in a year would have with particular PROMS, based on the evidence in the literature regarding observed associations with the level of disease activity in patients with FMF. Specifically, we predicted the following associations in children with FMF: functional status and QoL would be negatively associated, while the level of depressive symptoms and pain would be positively associated with the number of episodes in the past year.

## Materials and Methods

### Participants

Consecutive patients referred to the pediatric rheumatology outpatient clinics were recruited. Patients who had a diagnosis of FMF based on the pediatric FMF criteria or the Tel-Hashomer criteria were eligible (10, 11). All participating parents and children ≥ 8-year-old-age gave written informed assent and/or consent to participate in this study. The study protocol was approved by the institutional ethics committee.

#### Clinical Assessment

All patients were evaluated by a pediatric rheumatologist cross-sectionally. Face-to-face interviews were used to collect data on demographics (age, sex, age at diagnosis, consanguinity, and history of amyloidosis), treatment, mutations, and main clinical symptoms. The number of episodes in the past 12 months was obtained from patients or their parents. Then, patients were assigned into three groups by the number of FMF episodes: no episodes (Group 1), 1–4 episodes (Group 2), and more than 4 episodes (Group 3). Among this referred population, we included patients for whom we had data on three PROMs, the Pediatric Quality Life Inventory (PedsQL™) Generic Core Scale score, the Children’s Depression Inventory (CDI) score, and the Wong-Baker FACES^R^ pain rating scale (FACES^R^) score. In addition, to see if there are any differences among the patient groups, the elements of the AIDAI symptom scale were also collected (7). Medication adherence was assessed for medication-taking behavior, given that it is such an important confounder in research and a challenge in clinical care.

#### Genetic Screening

The QIAamp DNA mini kit (Qiagen, Germany) was used for DNA extraction. A reverse-hybridization method (Vienna Lab Diagnostics, Vienna, Austria) was performed for mutation analysis (12). Eleven variants in the *MEFV* gene were genotyped, such as E148Q, P369S, F479L, M680I (G > C and G > A), I692 del, M694V, M694I, K695R, V726A, A744S, and R761H.

### Patient Reported Outcome Measures

Patients completed the following patient reported outcome measures (PROMs) during the face-to-face interview during their clinical visit.

#### The PedsQL™ Generic Core Scale

Pediatric Quality Life Inventory, developed in 1999, by Varni et al. (13) is a short, standardized assessment tool to evaluate children with chronic diseases according to patients’ and parents’ perceptions of health-related quality of life (HRQoL). In 2005, the reliability and validity of the Turkish version of PedsQL were reported (14, 15). The PedsQL Generic Core Scale includes two summary scores with four scales and 23 items: the Physical Health Summary Score consisting of a physical functioning scale (8 items) and the Psychosocial Health Summary Score consisting of emotional functioning (5 items), social functioning (5 items), and school functioning (5 items) scales. Higher scores indicate better HRQoL. In this study, both parents and children reported the PedsQL.

#### Children’s Depression Inventory

Kovack’s CDI (16) is a 27-item self-report questionnaire to assess depressive symptoms experienced in the past 2 weeks in children 7–17 years old. The validated and reliable Turkish version was published in 1991 (17). Higher scores indicate the necessity to refer the patient for further evaluation in terms of clinical depression. In our research, both children and their parent(s) completed the CDI. Higher scores indicate more depressive symptoms.

#### Wong-Baker FACES^R^ Pain Rating Scale (FACES^R^)

The Wong-Baker FACES Pain Rating Scale (FACES) was developed for children to communicate about their pain (18). The FACES scale uses six hand-drawn, gender-neutral faces depicting smiling (0) to crying (10) placed at equal intervals horizontally: 0 (no hurt) and 10 (hurts worst). In this research, pain experienced by children < 8 years of age was evaluated by their parents. Higher scores indicate more pain.

#### Autoinflammatory Disease Activity Index

The AIDAI symptom scale includes 12 variables: fever, overall symptoms, abdominal pain, nausea/vomiting, diarrhea, headache, chest pain, painful nodes, arthralgia, or myalgia, swelling of the joints, eye manifestations, and skin rash. The calculation of the score is based on simple math; the sum of all 12 variables was divided by the number of months over which the diary was completed (0–372 in a month of 31 days). For the purposes of the current study, all the elements of the AIDAI symptom scale were asked for the last 1 year to the patients and their parents during the face-to-face interview. Instead of calculating the scores, we compared the elements of the AIDAI among the groups and hypothesized that the difference in the disease activity is mainly generated by the frequency of episodes. To test this hypothesis, we empirically chose to score dichotomously each element of the AIDAI depending on the presence or the absence of the individual item.

### Statistical Analysis

Statistical analysis was performed using the Statistical Package of Social Science (SPSS) for Windows, version 26.0 (SPSS Inc., Chicago, IL). Descriptive statistics were presented as frequencies and percentages for categorical variables and median [interquartile range (IQR)] for continuous variables as appropriate. The variables were investigated using visual (histograms and probability plots) and analytical methods (Kolmogrow–Simirnov test) to evaluate the normal distribution. The chi-squared test or Fisher’s exact test was used to compare categorical variables where appropriate. The Kruskal–Wallis test was used to compare non-normally distributed variables in the three groups. The Mann–Whitney *U*-test was performed to test the significance of pairwise differences using the Bonferroni correction to adjust for multiple comparisons. Concurrent validity was assessed using the associations of three PROMS with the number of FMF episodes in the past 12 months using the Spearman rank correlation coefficient (ρ). Correlation coefficients of ρ < 0.39 were considered as weak; 0.40–0.69 as moderate; and ≥ 0.70 as strong (19). A *p* < 0.05 was considered a statistically significant result.

## Results

### Participants

There were 270 patients enrolled in the registry. A total of 31 patients were excluded from the study because of incomplete response to PROMS, as mentioned in the Materials and Methods section. Forms were collected on 239 patients (44.4% men and 55.6% women) with FMF from seven different pediatric rheumatology centers in Turkey. A total of 176 forms were completed by parents and patients, and 63 forms were completed by parents who have children < 8 years old. There were 74 patients (31%) in the no FMF episode group (Group 1), 99 patients (41.4%) in the 1–4 episode group (Group 2), and 66 patients (27.6%) in ≥ 4 episode group (Group 3). The median age at the time of enrollment was 11 years (IQR: 7–14 years), the median age at disease onset was 3 years (IQR: 1–6 years), and the median age at diagnosis was 6 years (IQR: 3–9 years). Consanguinity, family history of FMF, and amyloidosis were reported as 29.7% (*n* = 71), 52.7% (*n* = 126), and 17.6 (*n* = 41), respectively. The three groups formed by the number of FMF episodes were similar according to age, age at diagnosis, gender, consanguinity, family history, and history of amyloidosis (*p* > 0.05), but the age at disease onset was significantly higher in Group 2 than the others (*p* = 0.001) (Table 1). Allele frequencies of the MEFV gene mutations in the groups were 60.4, 57.8, and 60% for M694V; 11.4, 15.6, and 12.1% for M680I; and 11.4, 7.8, and 9.9% for V726A, respectively (Table 1). The groups were similar in terms of M694V and V726A alleles (*p* = 0.843 and *p* = 0.46). Only the M680I allele was significantly more frequent in Group 2 (*p* = 0.01) (Table 1). The comparison of the genotypes for each group was also given in Table 1, and the groups were similar for homozygosity, compound heterozygosity, and heterozygosity (*p* = 0.94). Nearly all the patients (232 of 239 patients, 97.1%) were on colchicine treatment. The rest of the patients did not use colchicine because of intolerance (*n* = 2) and colchicine resistance (*n* = 5). Five patients were treated with biologic disease-modifying antirheumatic drugs (DMARDs) (2 Anakinra, 1 Canakinumab, and 2 Etanercept). Otherwise, medication adherence was similar among the groups.

**TABLE 1:** Demographic features and comparisons among the groups by the number of Familial Mediterranean fever (FMF) episodes.

	Group 1 (*n* = 74)	Group 2 (*n* = 99)	Group 3 (*n* = 66)	Total (*n* = 239)	*P*
Age, median (IQR) in years	12.0 (8.0–14.0)	11.0 (8.0–15.0)	10.0 (7.0–13.0)	11.0 (7.0–14.0)	0.156
Symptom onset age, median (IQR) in years	3.0 (1.0–5.0)	4.0 (1.0–7.0)	2.0 (1.0–5.0)	3.0 (1.0–6.0)	0.001*
Age at diagnosis, median (IQR) in years	5.0 (3.0–8.0)	7.0 (4.0–11.0)	6.0 (3.0–9.0)	6.0 (3.0–9.0)	0.06
Gender, *n* (%)					
Male	39 (52.7)	44 (44.4)	23 (34.8)	106 (44.4)	0.105
Female	35 (43.7)	55 (55.6)	43 (65.2)	133 (55.6)	
Consanguinity, *n* (%)	26 (35.1)	24 (24.2)	21 (31.8)	71 (29.7)	0.272
Family history, n (%)	32 (43.2)	54 (54.5)	40 (60.6)	126 (52.7)	0.108
History of amyloidosis, *n* (%)	12 (16.2)	19 (19.2)	11 (16.7)	41 (17.6)	0.856
Duration of episodes					
0–48 h	-	51 (61.4)	27 (49.1)	78 (56.5)	0.15
> 48 h	-	32 (38.6)	28 (50.9)	60 (43.5)	
**Allele frequency [*n* (%)]**					
M694V	64 (60.4)	74 (57.8)	51 (63.0)	189 (60.0)	0.843
M680I	12 (11.4)	20 (15.6)	6 (7.4)	38 (12.1)	0.01*
V726A	12 (11.4)	10 (7.8)	8 (9.9)	30 (9.5)	0.46
**Genotype [*n* (%)]**					
Homozygote	26 (40.6)	34 (41.0)	29 (46.8)	89 (42.6)	
Compound heterozygote	21 (32.8)	25 (30.1)	17 (27.4)	63 (30.1)	0.94
Heterozygote	17 (26.6)	24 (28.9)	16 (25.8)	57 (27.3)	

*No episode/year: Group 1, 1–4 episodes/year: Group 2, more than 4 episodes/year: Group 3.*

*1–4 episode group is significantly higher than others.

The characteristic signs and symptoms for each group are presented in Table 2. The most common symptoms reported were recurrent fever (92.1%), abdominal pain (91.6%), fatigue (79.9%), arthralgia (79.9%), leg pain (65.3%), myalgia (61.5%), headache (52.7%), and vomiting (51.9%). Groups were similar according to the clinical symptoms (*p* > 0.05) (Table 2). The groups were compared according to each item on the AIDAI symptom scale and there were no statistically significant differences among the groups (Table 2).

**TABLE 2:** The main clinical symptoms in line with the autoinflammatory disease activity index (AIDAI) symptom scale and their comparison among the groups by the number of FMF episodes.

	Group 2 (*n* = 99)	Group 3 (*n* = 66)	Total (*n* = 165)	p
	*n* (%)	*n* (%)	*n* (%)	
**Recurrent fever**	90 (90.9)	62 (93.9)	152 (92.1)	0.47
**Abdominal pain**	90 (90.9)	60 (90.9)	150 (90.9)	1.00
**Nausea-vomiting**	50 (50.5)	37 (56.1)	87 (52.7)	0.48
**Diarrhea**	30 (30.3)	26 (39.4)	56 (33.9)	0.22
**Headache**	49 (49.5)	42 (63.6)	91 (55.2)	0.07
**Chest pain**	43 (43.4)	30 (45.5)	73 (44.2)	0.79
**Arthralgia**	84 (84.8)	47 (71.2)	131 (79.4)	0.03
**Myalgia**	69 (69.7)	37 (56.1)	106 (64.2)	0.07
**Arthritis**	37 (37.4)	25 (37.9)	62 (37.6)	0.94
**Erysipelas like erythema**	10 (10.1)	8 (12.1)	18 (10.9)	0.68

*Group 2:1–4 episodes/year, Group 3: more than 4 episodes/year.*

### Patient-Reported Outcome Measures

The PedsQL median (IQR) score was 77.3 (65.2–84.1) according to parents and 78.4 (67.8–85.6) according to children. For parent and child PedsQL scale scores (physical and psychosocial functioning scales) and total scores, patients in Group 1 had higher scores that meant better HRQoL than Group 2, and Group 2 had higher scores (better HRQoL) than Group 3 (*p* < 0.05) (Table 3). The median (IQR) CDI score was 8.0 (4.0–12.0) according to parents and 8.0 (5.0–12.0) according to children. Patients in Group 2 had higher parent CDI scores than those in Group 1 (*p* < 0.001). Child CDI scores were significantly lower in Group 1 than Group 2 (*p* = 0.01), and in Group 2 than Group 3 (*p* = 0.03) (Table 4). The FACES median (IQR) score was 2.0 (0–6.0) according to both parents and children. Both parent and child FACES scores were significantly lower in Group 1 than in Group 2 (*p* < 0.001), and in Group 2 than in Group 3 (*p* < 0.001) (Table 3).

**TABLE 3:** The comparisons of the scores of PedsQL, FACES, and CDI among groups by the number of FMF episodes.

		a	b	c					
		Group 1	Group 2	Group 3	Total	a–b	a–c	b–c	
		Media*n* (IQR)	Media*n* (IQR)	Media*n* (IQR)	Media*n* (IQR)	*P*	*p*	*p*	*p*
PedsQL (parent)		*n* = 61	*n* = 89	*n* = 57	*n* = 207				

	Physical health	60.9 (53.1–62.5)	56.3 (43.7–62.5)	42.2 (31.3–59.4)	56.3 (40.6–62.5)	0.005	<0.001	<0.001	<0.001
	Psychosocial health*	91.7 (86.7–96.7)	83.4 (74.2–93.4)	73.4 (60.8–85.0)	85.0 (73.4–93.4)	<0.001	<0.001	0.001	<0.001
	Total score	83.3 (79.1–87.2)	75.9 (65.5–83.3)	65.9 (54.6–76.5)	77.3 (65.2–84.1)	<0.001	<0.001	<0.001	<0.001

PedsQL (child)		*n* = 56	*n* = 75	*n* = 41	*n* = 172				

	Physical health	60.9 (56.3–62.5)	56.3 (41.4–62.5)	42.2 (33.6–50.0)	56.3 (40.62–62.5)	0.001	<0.001	0.001	<0.001
	Psychosocial health*	93.4 (86.7–98.4)	81.7 (75.0–93.4)	78.4 (69.2–90.8)	86.7 (76.7–94.6)	<0.001	<0.001	0.89	<0.001
	Total score	84.9 (79.1–88.8)	75.5 (68.1–84.1)	68.1 (59.1–80.4)	78.4 (67.8–85.6)	<0.001	<0.001	0.02	<0.001

CDI (parent)		*n* = 63	*n* = 88	*n* = 55	*n* = 206				

		6.0 (3.0–9.0)	8.0 (4.0–12.0)	9.0 (6.0–15.0)	8.0 (4.0–12.0)	0.054	<0.001	0.09	<0.001

CDI (child)		*n* = 55	*n* = 75	*n* = 42	*n* = 172				

		7.0 (2.0–10.0)	8.0 (6.0–11.0)	11.5 (6.75–16.0)	8.0 (5.0–12.0)	0.011	<0.001	0.03	<0.001

FACES (parent)		*n* = 74	*n* = 99	*n* = 66	*n* = 239				

		0 (0–2.0)	0 (0–6.0)	6 (0–8.0)	2 (0–6.0)	0.006	<0.001	<0.001	<0.001

FACES (child)		*n* = 58	*n* = 76	*n* = 42	*n* = 176				

		0 (0–2.0)	2 (0–6.0)	6 (4.0–8.0)	2 (0–6.0)	0.008	<0.001	<0.001	<0.001

*No episode/year: Group 1, 1–4 episodes/year: Group 2, more than 4 episodes/year: Group 3, PedsQL, Pediatric Quality Life Inventory; CDI, Children’s Depression Inventory; FACES, Wong-Baker FACES Pain Rating Scale.*

*Representing summary of emotional, social, and school subscales.

**TABLE 4:** Results of Spearmen’s correlations between the number of episodes and PROMs.

	PROM	Episode groups-Spearman correlation (ρ)	95% confidence interval		*p*-value
			Lower	Upper	
Parents	PedsQL total score	–0.48	–0.58	–0.35	<0.001
	PedsQL physical health score	–0.44	–0.54	–0.31	<0.001
	PedsQL psychosocial health score	–0.44	–0.55	–0.31	<0.001
	CDI	0.27	0.13	0.40	<0.001
	FACES	0.47	0.35	0.57	<0.001
Children	PedsQL total score	–0.44	–0.56	–0.30	<0.001
	PedsQL physical health score	–0.45	–0.57	–0.33	<0.001
	PedsQL psychosocial health score	–0.39	–0.52	–0.24	<0.001
	CDI	0.30	0.15	0.44	<0.001
	FACES	0.45	0.32	0.57	<0.001

*PROM, Patient-reported outcome; PedsQL, Pediatric Quality Life Inventory; CDI, Children’s Depression Inventory; FACES, Wong-Baker FACES Pain Rating Scale.*

### Concurrent Validity

According to PROMs completed by parents, moderate correlations were found between the number of episodes and PedsQL total score (ρ = −0.48; 95% *CI* = −0.58 to −0.35, *p* < 0.001), CDI (ρ = 0.27; 95% *CI* = 0.13–0.40, *p* < 0.001) and between the number of episodes and FACES score (ρ = 0.47, 95% *CI* = 0.35–0.57, *p* < 0.001). In addition, there were moderate correlations between the number of episodes and PROMs completed by children themselves (Table 4).

## Discussion

Assessment of disease activity in patients with AIDs remains a challenge due to the nature of these disorders, such as an episodic course, phenotypic differences, and a low likelihood of being able to see the patient during an acute episode. We have provided preliminary results suggesting that the number of episodes may offer a valid measure of disease activity in patients with FMF that can be easily administered in a clinic.

The european alliance of associations for rheumatology (EULAR) consensus recommendations for the treatment of FMF are that those patients should be seen two times a year (3). For the management of AIDs, it is mandatory to evaluate the disease activity (2). Hence, an international group of experts developed an activity score to assess the disease activity, called AIDAI, for four major hereditary periodic fever syndromes, such as FMF. It has not been widely adopted, however, since it is not practical for use in clinical trials or practice (9). Since it is not feasible to expect families to complete a prospective diary for the long period between clinical appointments, it is important to have a valid measure of disease activity that is easy to administer during clinic visits.

The AIDAI is a valid and reliable patient diary to assess the disease activity in four major hereditary syndromes. According to the AIDAI, a 3-month period is more suitable for surveying FMF, and it is not easy and applicable for defining the disease activity in FMF, especially if the patient is experiencing fewer episodes during the year. In the AIDAI Consensus Conference, the experts agreed that fever, joint symptoms, serositis, chest and skin symptoms, and the number and duration of episodes were all important in the evaluation of disease activity for patients with FMF (7). AIDAI consists of 12 disease-related symptoms and the calculation of the score is based on simple math; the sum of all 12 variables divided by the number of months over which the diary was completed (0–372 in a month of 31 days). In terms of those criteria, there were no differences among the groups in our cohort except for the number of episodes. On the basis of our results, the number of episodes itself alone is a valid indicator of disease activity in patients with FMF.

Patients with AIDs should also be followed with clinical evaluation, laboratory test, QoL, tolerance, and treatment adherence. Relationships of patient-parent reported outcomes with disease activity have been studied in juvenile idiopathic arthritis (JIA) (20–26), juvenile idiopathic myositis (23), rheumatoid arthritis (27–29), systemic lupus erythematosus (SLE) (30–35), Behcet disease (36), and ankylosing spondylitis (AS) (34, 37). The data on the relationship between disease activity and patient-parent reported outcomes in FMF are very limited. In the present study, PedsQL, CDI, and FACES scores were used as patient-parent reported outcomes.

Quality of life is an important factor in determining disease status and defining and evaluating the effects of management strategies (38). The concurrent validity of the number of episodes was assessed using PedsQL and a moderate correlation was found, which is not surprising in our study. PedsQL scores differed across groups, decreasing when the number of episodes increased in our cohort. Buskila et al. showed that the QoL in patients with FMF is inversely correlated with the number of FMF episodes for a year (39). Alayli et al. compared the HRQoL between patients with FMF and healthy people and indicated that the QoL is impaired in children with FMF (40). Sahin et al. have reported no relationship between QoL and the number of episodes in adult patients using SF-36 (41). On the contrary, the studies conducted with FMF patients during their episode-free period, reported that QoL is even better in patients with FMF compared with healthy controls (42). Taken all these together, it is clear that the number of episodes is inversely correlated with HRQoL in patients with FMF.

The CDI scores were higher in groups with more episodes in our study population. Makay et al. reported that the CDI scores of children and adolescents with FMF were significantly higher than those of a healthy control group (43). Sonmez et al. evaluated depression by using CDI in patients (remission) with FMF and healthy controls and found no difference between patients and healthy controls (44). It was reported in adults that depression was more frequent in patients with FMF than in healthy controls using the Hospital Anxiety and Depression Scale (HADS) (45, 46) and the Hamilton Depression Scale (HDS) (47). Our study is the first one that compared the CDI scores according to the number of episodes.

We used FACES as a pain scale in our cohort, and the results showed that pain scores were similarly higher with more episodes reported. We showed that worse health-related QoL (PedsQL) and increased depression (CDI) were correlated with the number of episodes.

The limitations of the present study need to be mentioned. One of the major limitations of our study is that we were not able to analyze the AIDAI score because of poor patient and parent adherence. On this occasion, we have faced the difficulty of using AIDAI in a large patient cohort, especially in adolescents. Another limitation that needs to be mentioned is that the current study was designed as cross-sectional and it is important to see the changes with PROMs, also prospectively, to complete all validation steps. We just performed concurrent validity due to the nature of the current study.

## Conclusion

In conclusion, the present study showed that a larger number of episodes are related to worse patient- and parent-reported outcomes. The PedsQL, CDI, and FACES scores were significantly different among the groups divided according to the number of FMF episodes in a year, in a homogeneous study population in terms of demographic features, mutation types, clinical symptoms, and treatment adherence. This study showed that the number of episodes is the key element of disease activity in patients with FMF and can be used on its own to assess disease activity.

## Data Availability Statement

The raw data supporting the conclusions of this article will be made available by the authors, without undue reservation.

## Ethics Statement

The studies involving human participants were reviewed and approved by Clinical Trials Ethics Committee of GMMA, Ankara, Turkey. Written informed consent to participate in this study was provided by the participants’ legal guardian/next of kin.

## Author Contributions

DP and KS: data analysis and interpretation. DK, NA, BM, YB, HP, and OK: collecting patient data and providing clinical information. DP, ZA, and MR: writing—original draft preparation. DP, KS, ZA, MR, RL, RB, and ED: writing—review and editing. ED: as a PI had full access to all the data in the study and takes responsibility for the integrity of the data and the accuracy of the analysis. All authors have read and agreed to the published version of the manuscript.

## Conflict of Interest

The authors declare that the research was conducted in the absence of any commercial or financial relationships that could be construed as a potential conflict of interest.

## Publisher’s Note

All claims expressed in this article are solely those of the authors and do not necessarily represent those of their affiliated organizations, or those of the publisher, the editors and the reviewers. Any product that may be evaluated in this article, or claim that may be made by its manufacturer, is not guaranteed or endorsed by the publisher.
